# How I do It: Endoscopic transorbital resection of sphenoid osseous meningioma via the lateral orbital ‘sliding coach door’ approach

**DOI:** 10.1007/s00701-025-06484-w

**Published:** 2025-03-20

**Authors:** James M. W. Robins, Jiten Parmar, Asim J. Sheikh

**Affiliations:** 1 Department of Neurosurgery, G Floor, Leeds General Infirmary, LEEDS, Jubilee Building, LS1 3EX London, UK; 2 Department of Oral and Maxillofacial Surgery, Leeds General Infirmary, LEEDS, Great George St, LS1 3EX London, UK

**Keywords:** Endoscope, Transorbital, Meningioma

## Abstract

**Background:**

A 63-year-old presented with reduced left visual acuity and V1 sensation. Imaging demonstrated left sphenoid osseous meningioma narrowing superior orbital fissure with intracranial extension to superior temporal gyrus.

**Method:**

Endoscopic transorbital approach utilising novel lateral orbit ‘sliding coach door’ osteotomy performed. Lateral canthal incision with lateral canthal ligament division mobilises and decompresses globe infero-medially. Osteotomy performed, tethered by temporalis. Osteotomy slides postero-laterally creating working space lateral to inferior and superior orbital fissures.

**Conclusion:**

This technique requires reduced soft tissue dissection and facilitates reconstruction. Adequate working space enabled satisfactory resection with residual dural tail requiring future surveillance. Cosmesis was satisfactory.

**Supplementary Information:**

The online version contains supplementary material available at 10.1007/s00701-025-06484-w.

## Relevant surgical anatomy

**Fig. 1:**
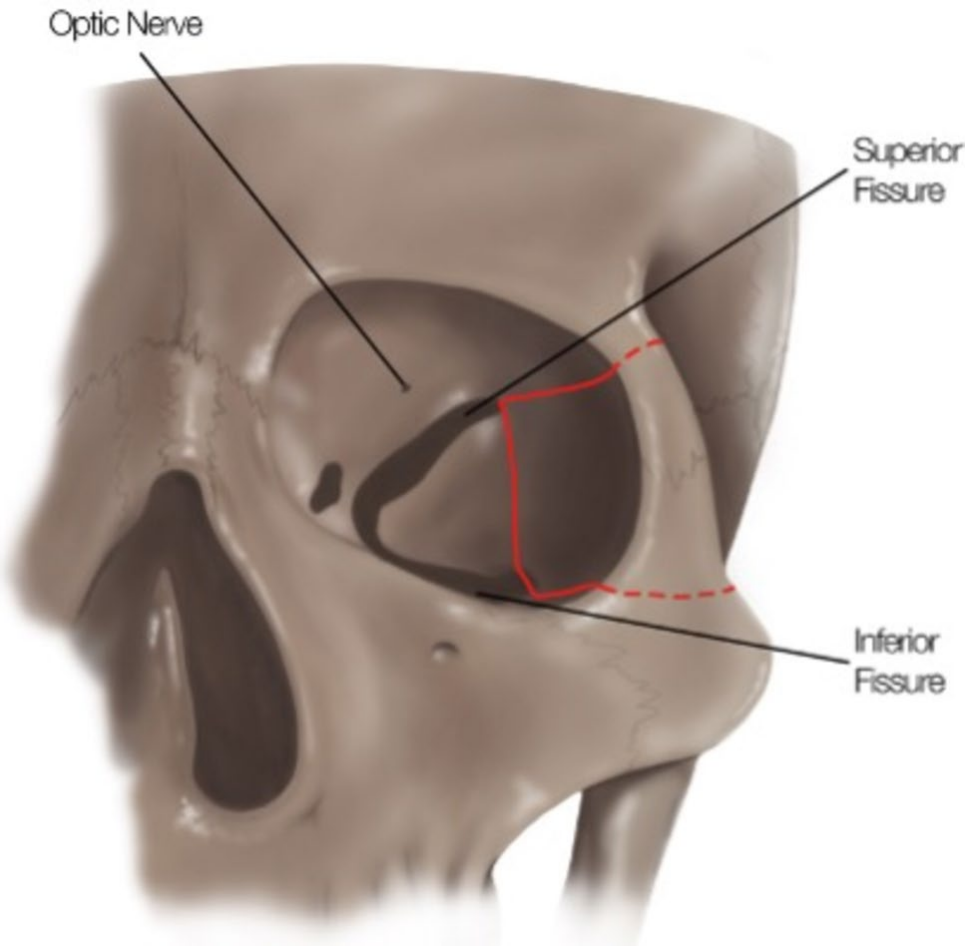
Demonstration of the key structures to identify pre-operatively including superior and inferior orbital fissure

Understanding of orbital anatomy is fundamental to successful execution of this technique without complication. The key structures to identify pre-operatively include the optic canal, superior and inferior orbital fissures (Fig. 1). Intraoperative identification of these fissures is crucial prior to resection of the lateral posterior orbital wall.

## Description of technique

### Scans and navigation

**Fig. 2:**
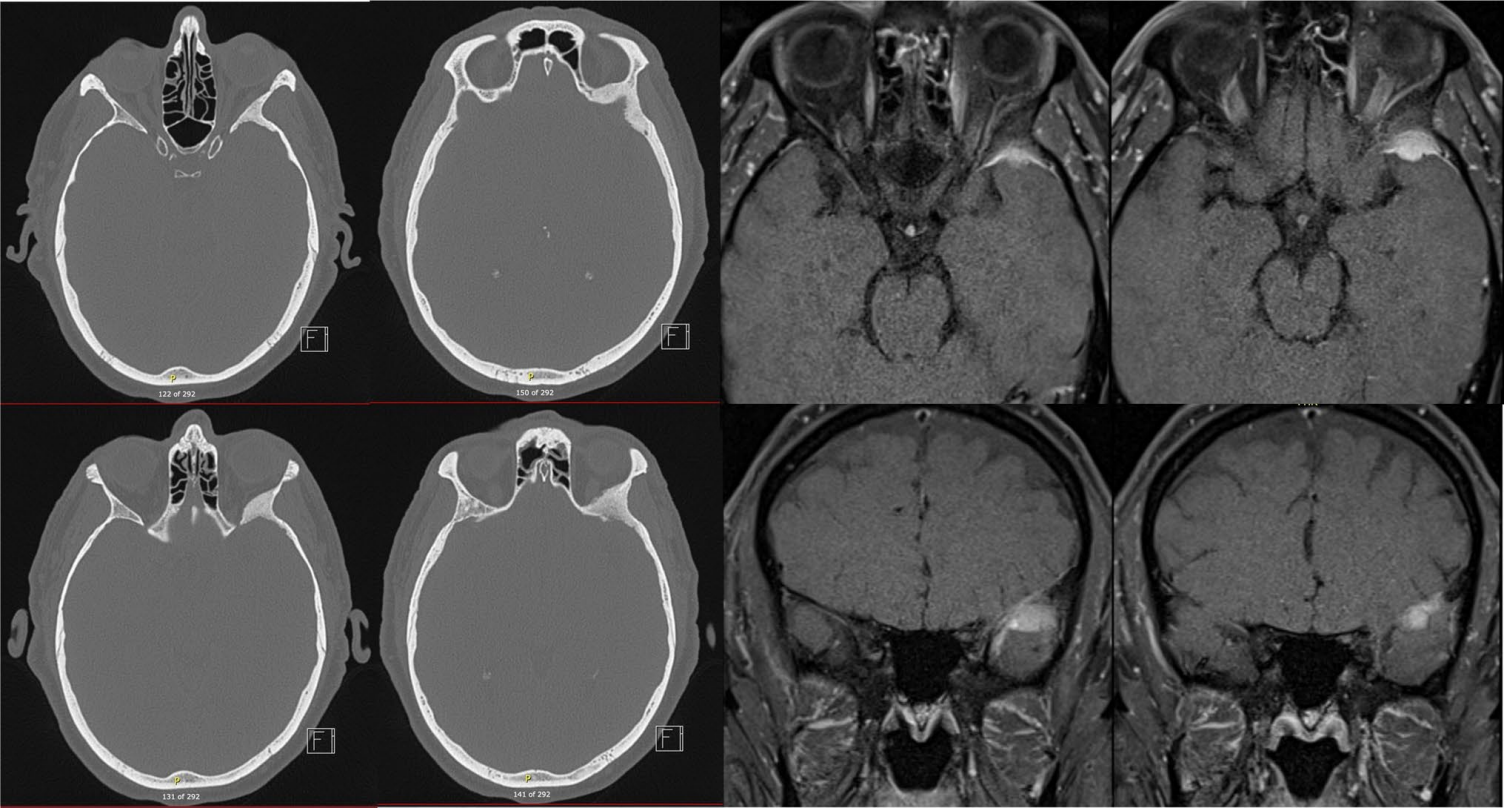
Pre-operative CT and MRI T1 with gadolinium administration demonstrating left sphenoid osseous meningioma and temporal meningioma

**Fig. 3:**
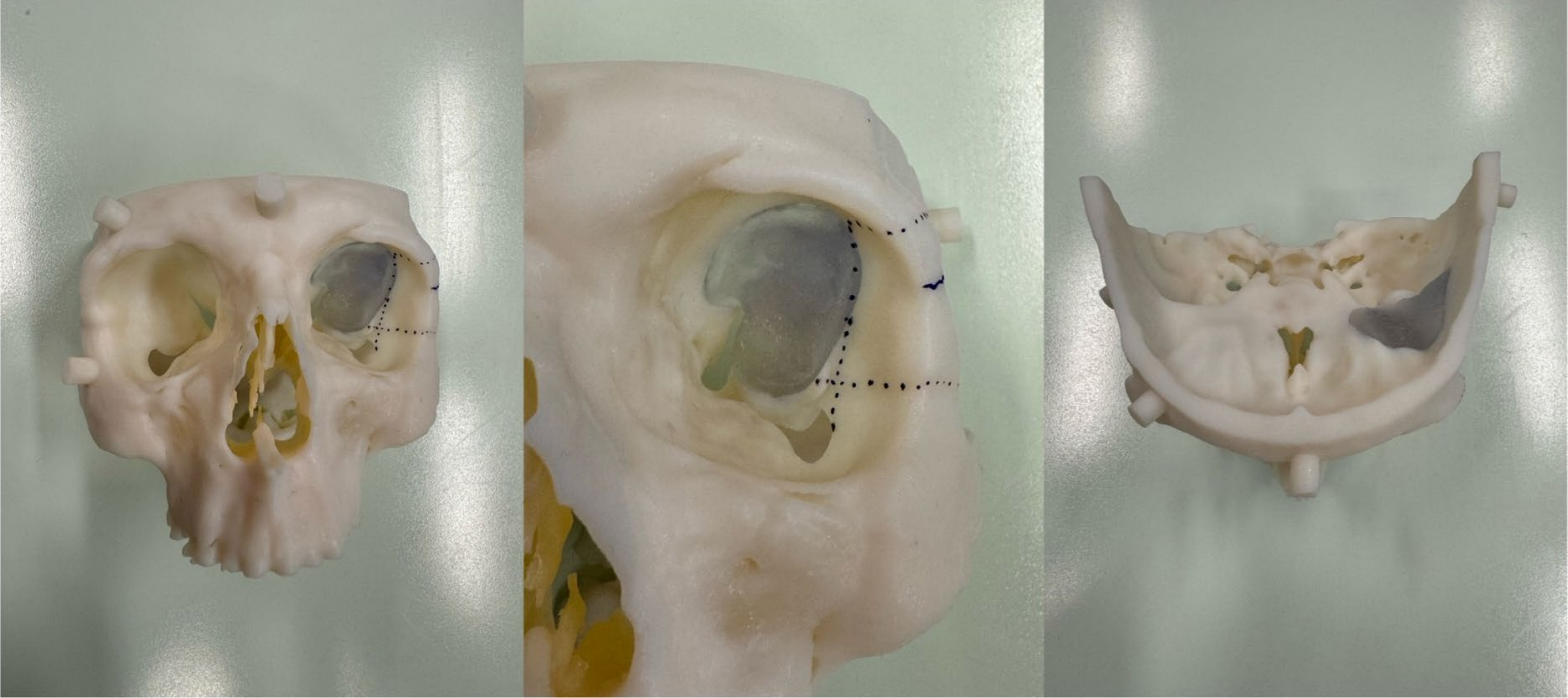
Pre-operative 3D modelling demonstrating area infiltrated with tumour (grey) for removal and the bony cuts for the osteotomy (dotted lines)

Pre-operative tri-planar MRI scan with orbital and brain tumour protocol – T2, T1 pre and post gadolinium sequences with fine cut (0.6 mm) CT is performed (Fig. 2). Pre-operative 3D modelling is also performed (Fig. 3). These investigations enable multidisciplinary discussion for optimum surgical planning. Optical navigation (BrainLab AG, Munich, Germany) is utilised.

### Anaesthesia

Total intravenous anaesthesia (TIVA) is performed to enable titratable and responsive anaesthesia with strict blood pressure control for haemostasis control. Extubation with minimal coughing, combined with gentle orbital pressure on anaesthesia emergence prevents compromise of reconstruction.

### Positioning

**Fig. 4:**
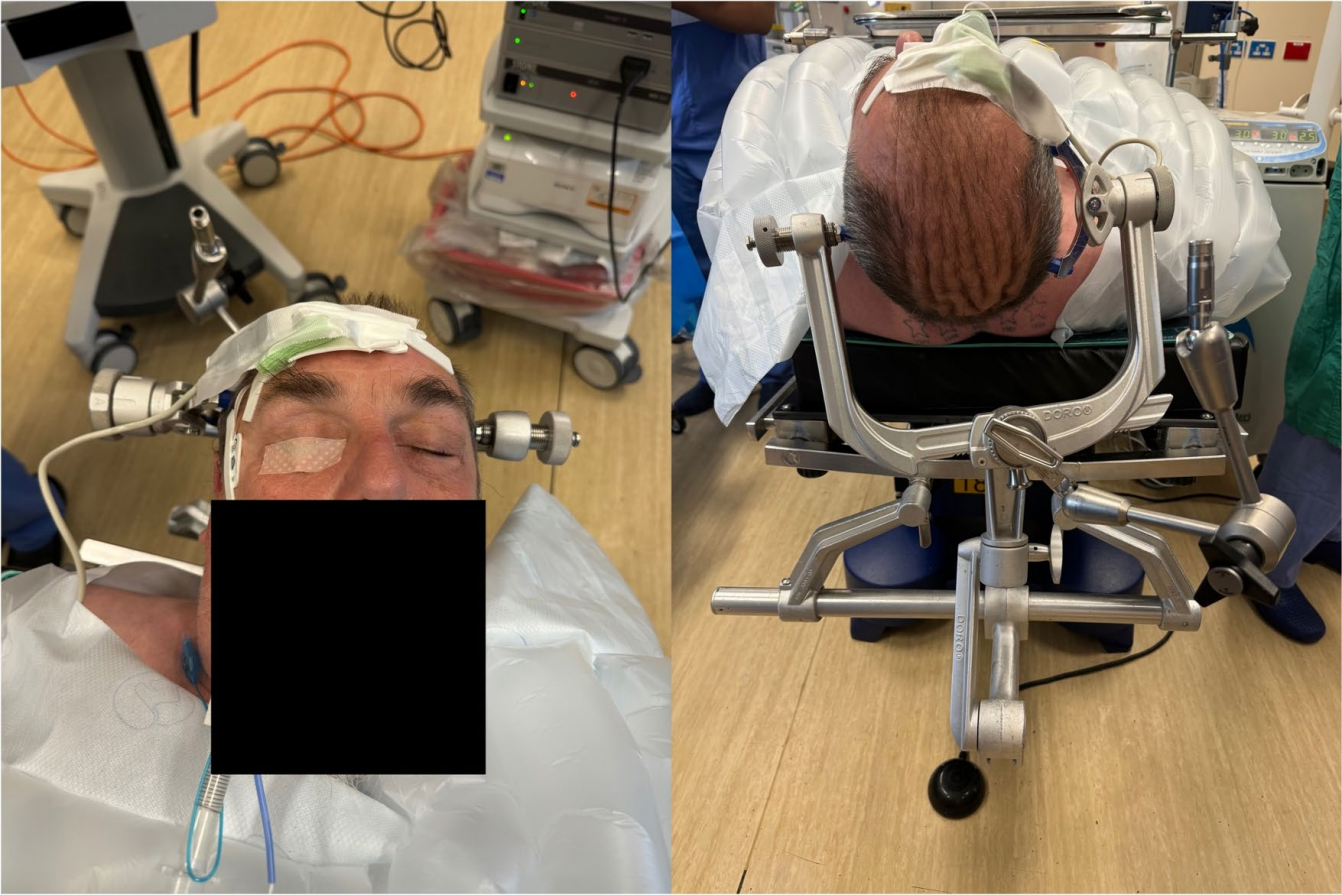
Patient positioning – supine, cranial pins, head slightly rotated to left with optical navigation

The patient is positioned supine in cranial pins with head slightly rotated to the left and elevated to 15° (Fig. 4).

### Technique

Patient is positioned and optical navigation registered.

After aqueous iodine preparation, an eye shield is placed, local anaesthetic infiltrated (2% lidocaine with 1:80 000 adrenaline) and lateral canthal skin incision performed.

The lateral canthal ligament is divided, conjunctiva incised and dissection to bone to develop a subperiosteal plane.

The globe is medialised and periorbital contents are elevated from the lateral orbital wall with cauterisation of emissary vessels[1, 3].

The inferior orbital fissure is visualised.

The lateral orbital osteotomy is performed with confirmation of the depth intraoperatively using a ruler.

Frequent pupillary checks are performed throughout, minimum every 30 min.

The osteotomy is pre-plated with low profile titanium plates to aid reconstruction.

Fixed retraction is prepared for osteotomy retraction.

The orbital cuts for the osteotomy are performed as demonstrated on the preoperative model. The osteotomy is completed with a pterygoid osteotome.

**Fig. 5:**
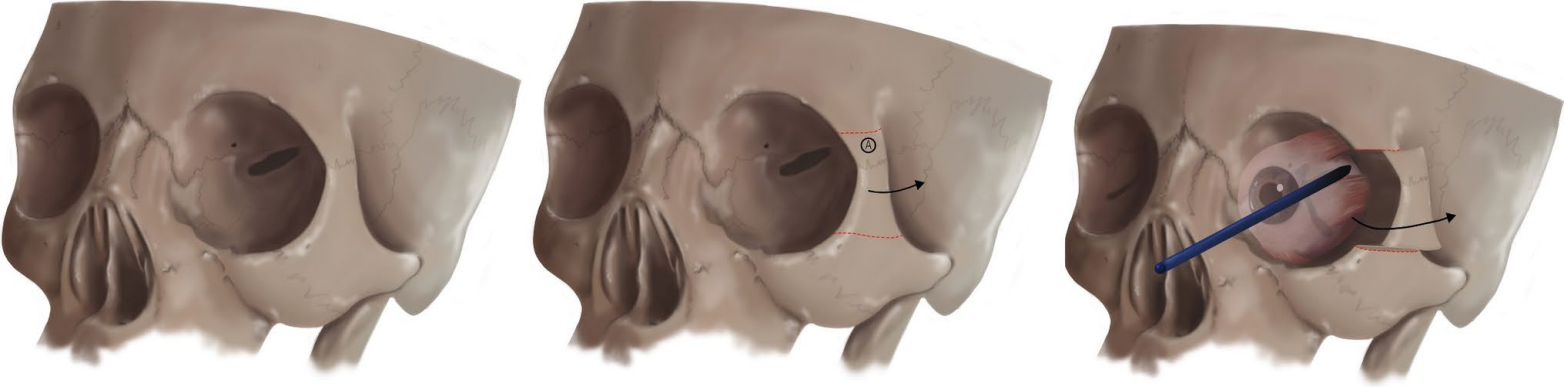
Demonstration of the lateral ‘sliding coach door’ osteotomy with mobilisation of the lateral orbital wall laterally and posteriorly Credit: Colin Sullivan, Medical Illustrator, Leeds General Infirmary

The osteotomy is mobilised and slid laterally and posteriorly similar to a coach door providing a surgical access corridor (Fig. 5).

The sphenoid osseous meningioma lateral to the inferior and superior orbital fissures is removed with a coarse diamond burr [2–5].

Greater wing of sphenoid is removed with upcuts to expose the temporal dura and dura is cauterised prior to durotomy.

The intracranial meningioma is mobilised, dissected free and removed. Dural edges are cauterised, and underlying anterior temporal lobe is inspected for injury.

The defect is reconstructed with inlay dural substitute, dural sealant, onlay dural substitute, dural sealant and fibrin glue followed by osteotomy plating.

The globe is assessed after reconstruction followed by closure of the skin, but not conjunctiva to prevent orbital compartment syndrome.

Eye movements are confirmed manually via the recti muscles and the immediate postoperative appearances are demonstrated.

The patient is administered a STAT dose of dexamethasone and 5 days of antibiotic prophylaxis.

**Fig. 6:**
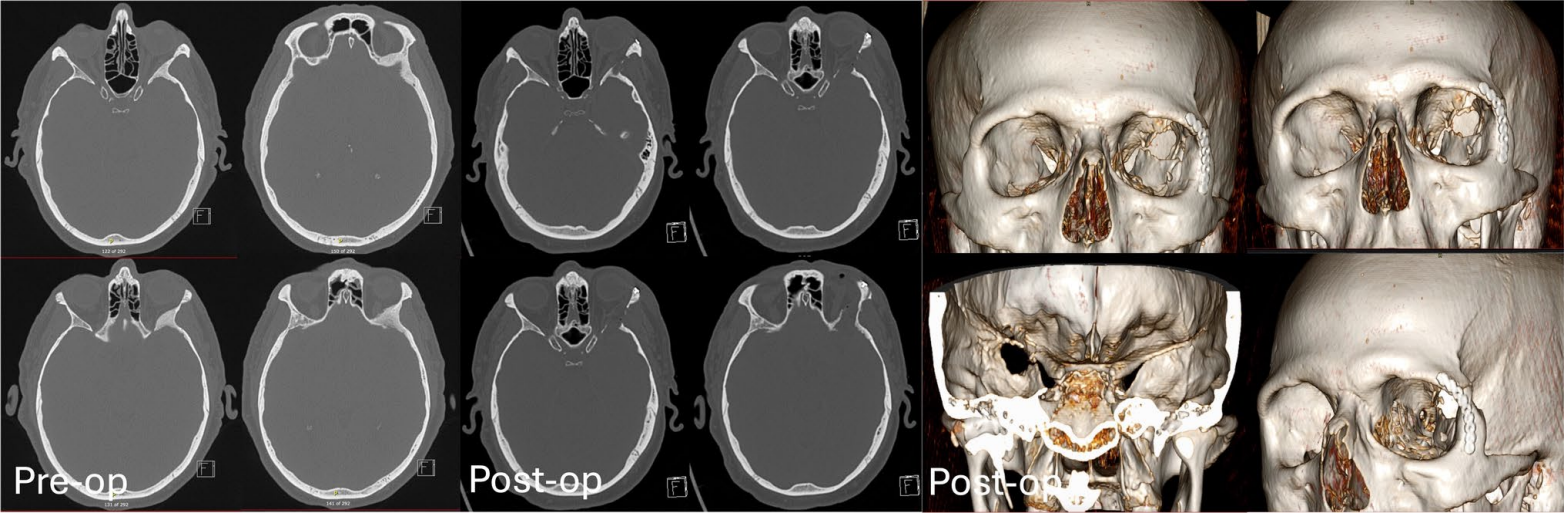
Pre- and post-operative CT with bony reconstructions demonstrating bony removal and tumour resection

Day 1 post-operative CT is performed to demonstrate decompression of the superior orbital fissure, tumour resection and reconstruction (Fig. 6).

**Fig. 7:**
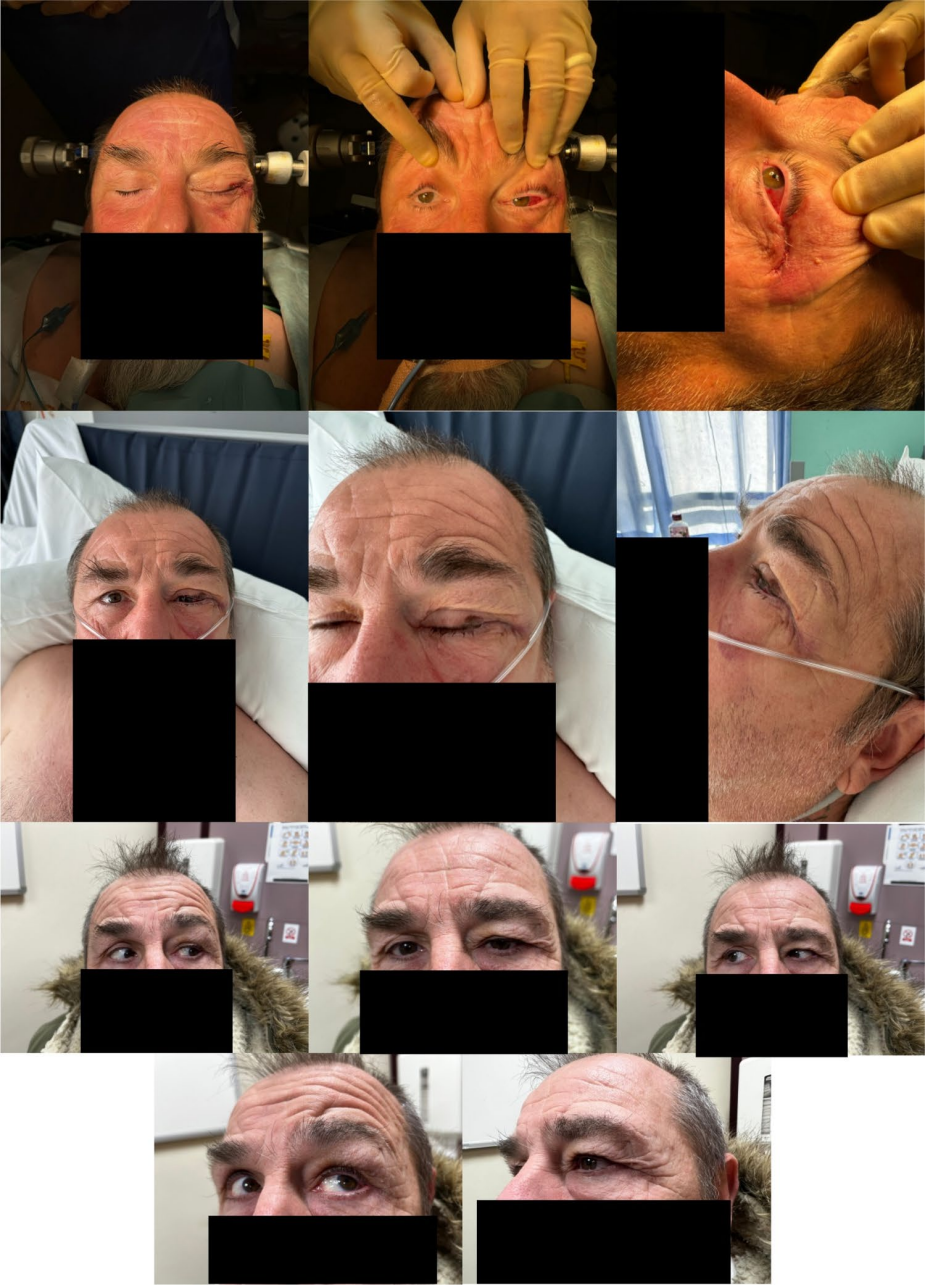
Immediate, Day 1 and 3 months post-operative photographs demonstrating satisfactory cosmetic result

**Fig. 8:**
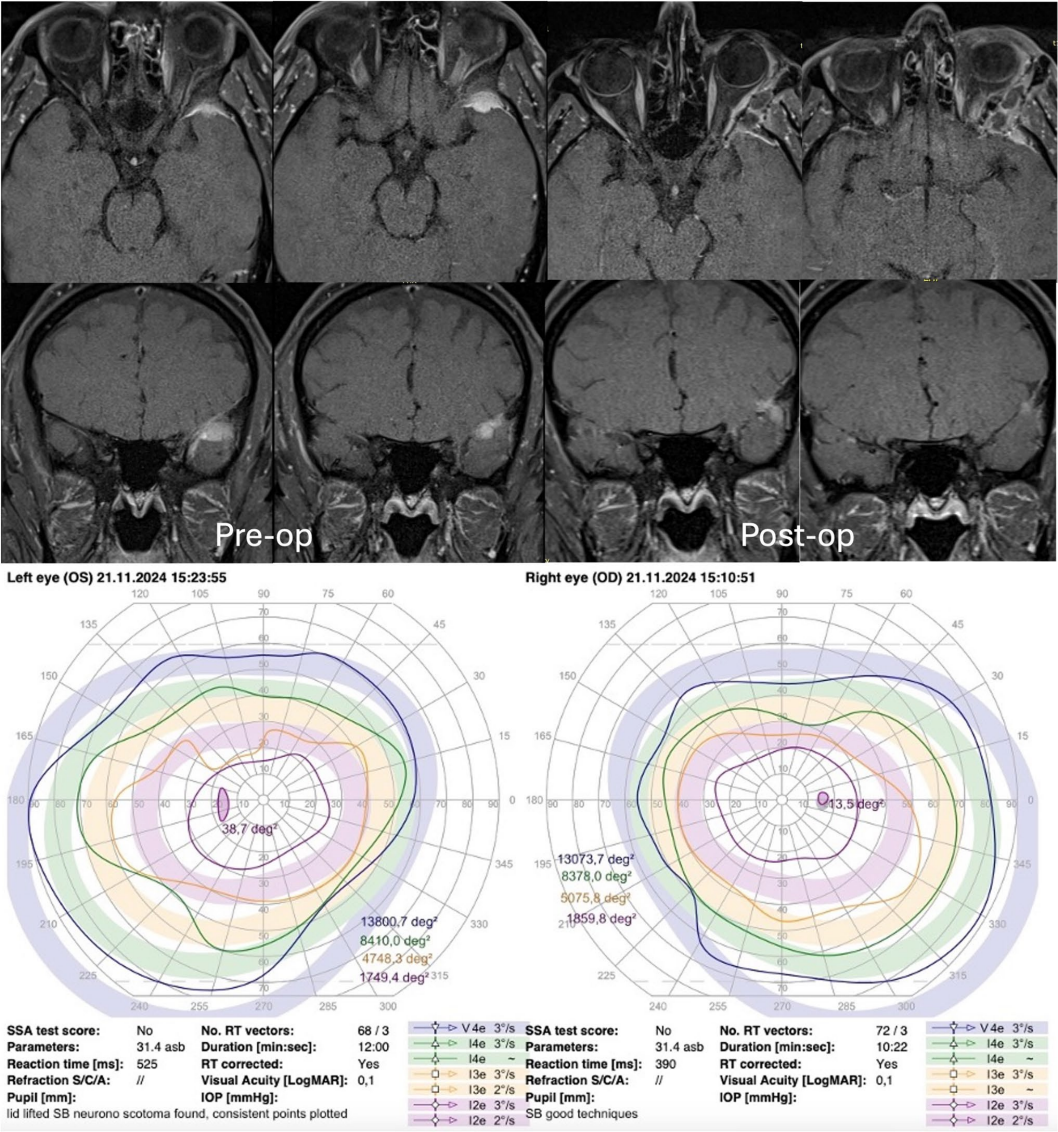
Pre- and post-operative MR T1 with gadolinium demonstrating tumour resection with post-surgical changes with suspicion of a dural tail requiring future surveillance imaging. Satisfactory Goldmann visual field assessment at 2 months post-operatively

Post-operative photographs are acquired to demonstrate a satisfactory cosmetic result (Fig. 7) and, post-operative MRI and visual fields are performed to assess for tumour resection and visual field loss (Fig. 8).

The patient recovered well with resolution of V1 numbness, stable visual acuity, normal eye movements and was discharged home on post-operative day 3.

## Indications

This minimally invasive technique reduces tissue dissection and avoids brain retraction, minimising risk of injury.

Indications include:

- Spheno-orbital tumours lateral to the superior and inferior orbital fissures with no lateral extension into temporalis, temporal squama or lateral dural tail.
- Anterior temporal and cavernous sinus lesions

## Limitations

- There is a learning curve with all trans-orbital techniques.
- When performing this minimally invasive trans-orbital technique, careful pre-operative assessment of CT and MR imaging is essential as this technique cannot be used to access orbital roof, anterior clinoid process, temporal muscle and temporal squama. In these situations, alternative trans-orbital/nasal approaches may be more appropriate or open techniques, particularly in the case of en plaque meningioma.
- In this case, this novel technique limited lateral access to tumour which can make resection of dural tail difficult.

## How to avoid complications

- Preoperative 3D modelling significantly enhances understanding of the approach and tumour location in relation to critical structures (Fig. 2). When combined with a multidisciplinary surgical team, in this case maxillo-facial surgeon with significant orbital experience, complications can be anticipated and avoided.
- Use of an eye shield is crucial to protect the cornea.
- Frequent pupillary checks every 30 min and dialogue with anaesthetist on incidence of bradycardia alerts to excessive orbital pressure and risk of visual loss. The division of the lateral canthal ligament in this technique also mobilises and decompresses the globe to further mitigate risk of injury.
- Pre-plating the lateral orbital wall before osteotomy aids satisfactory reconstruction cosmesis.

## Specific information for patient

Patients need to be aware for the following:

- This is a minimally invasive approach with limited indications and limitations.
- There is also a learning curve associated with trans-orbital procedures in general. As such there is a small but important risk to local structures resulting in facial numbness, loss of eye movements and visual loss.

## Ten key points summary

1. This is a minimally invasive approach which enables removal of selected tumours which do not extend into the orbital roof, anterior clinoid process, temporalis or temporal squama.
2. Pre-operative 3D modelling is a useful adjunct to determine tumours amenable to this technique and location relative to critical local structures.
3. There is a learning curve associated with trans-orbital approaches.
4. Multidisciplinary surgical team including surgeons familiar with orbital operating e.g. maxillofacial surgeons, minimises risk of complications.
5. Division of the lateral canthal ligament enables globe mobilisation preventing globe injury.
6. Leaving lateral wall osteotomy attached to temporalis reduces tissue dissection, facilitates reconstruction whilst providing adequate surgical corridor.
7. Non closure of the conjunctiva prevents orbital compartment syndrome.
8. Optimised anaesthesia limits intraoperative bleeding, prevents extubation coughing to protect reconstruction.
9. This procedure is well tolerated and results in good cosmetic outcome.
10. Limitations of this approach include limited lateral access for chasing dural tail and alternative techniques should be considered.

## Supplementary Information

Below is the link to the electronic supplementary material.

ESM 1 (MP4 135 MB)

## Data Availability

No datasets were generated or analysed during the current study.
